# Machine learning for the prediction of acute kidney injury in critical care patients with acute cerebrovascular disease

**DOI:** 10.1080/0886022X.2022.2036619

**Published:** 2022-02-15

**Authors:** Xiaohong Zhang, Siying Chen, Kunmei Lai, Zhimin Chen, Jianxin Wan, Yanfang Xu

**Affiliations:** Department of Nephrology, the First Affiliated Hospital, Fujian Medical University, Fuzhou, China

**Keywords:** Acute kidney injury, acute cerebrovascular disease, machine learning methods, risk prediction models

## Abstract

**Purpose:**

Acute kidney injury (AKI) is a common complication and associated with a poor clinical outcome. In this study, we developed and validated a model for predicting the risk of AKI through machine learning methods in critical care patients with acute cerebrovascular disease.

**Methods:**

This study was a retrospective study based on two different cohorts. Five machine learning methods were used to develop AKI risk prediction models. We used six popular metrics (AUROC, F2-Score, accuracy, sensitivity, specificity and precision) to evaluate the performance of these models.

**Results:**

We identified 2935 patients in the MIMIC-III database and 499 patients in our local database to develop and validate the AKI risk prediction model. The incidence of AKI in these two different cohorts was 18.3% and 61.7%, respectively. Analysis showed that several laboratory parameters (serum creatinine, hemoglobin, white blood cell count, bicarbonate, blood urea nitrogen, sodium, albumin, and platelet count), age, and length of hospital stay, were the top ten important factors associated with AKI. The analysis demonstrated that the XGBoost had higher AUROC (0.880, 95%CI: 0.831–0.929), indicating that the XGBoost model was better at predicting AKI risk in patients with acute cerebrovascular disease than other models.

**Conclusions:**

This study developed machine learning methods to identify critically ill patients with acute cerebrovascular disease who are at a high risk of developing AKI. This result suggested that machine learning techniques had the potential to improve the prediction of AKI risk models in critical care.

## Introduction

Acute kidney injury (AKI) is a common syndrome that is characterized by a sudden reduction in kidney function and is caused by different etiologies. AKI is a serious complication in intensive care units (ICUs) and can increase the mortality rate for critically ill patients. Previous studies have reported that the incidence of AKI in ICU patients ranges from 19.2% to 57.3% [1,2]. However, despite significant advances in health care, the incidence of AKI is still increasing [1,3–5]. Although many studies have described the risk factors and clinical outcomes of AKI [5–7], we still know very little about the risk of AKI in patients with acute cerebrovascular disease.

In general, acute cerebrovascular disease includes both hemorrhagic stroke and ischemic stroke. The acute cerebrovascular disease has a high mortality rate if patients are complicated by AKI. A previous study showed that the incidence of AKI among an eastern European population with ischemic stroke and hemorrhagic stroke was 14.5% [8]. A meta-analysis further showed that the incidence of AKI after stroke was 9.61% and that the risk of AKI after hemorrhagic stroke was much higher at 20% [9]. Other studies have also shown that AKI is associated with increased mortality, disability, and a poorer neurological outcome [10–12]. It is important to identify patients at risk of AKI so that we can prevent the development of AKI and improve patient outcomes.

The development of artificial intelligence has led to a significant improvement in the predictive models used for estimating the risk of AKI [13–16]. There was a study using a Bayesian networks (BNs) model to predict the risk of AKI in gastrointestinal cancer (GI) patients. BNs model achieved good predictive capacity compared with other machine learning models [17]. However, there is still limited data relating to the AKI risk prediction models using machine learning methods in patients with acute cerebrovascular disease in the ICU setting. In the present study, we used a public database (the Medical Information Mart for Intensive Care (MIMIC)-III database) with the database held by our local hospital, to build a range of models for predicting the risk of AKI in critically ill patients with acute cerebrovascular disease. In generating our models, we applied a machine learning approach to develop a clinical risk model that could assist clinicians with decision-making and clinical management.

## Methods

### Data source

**Figure 1.**
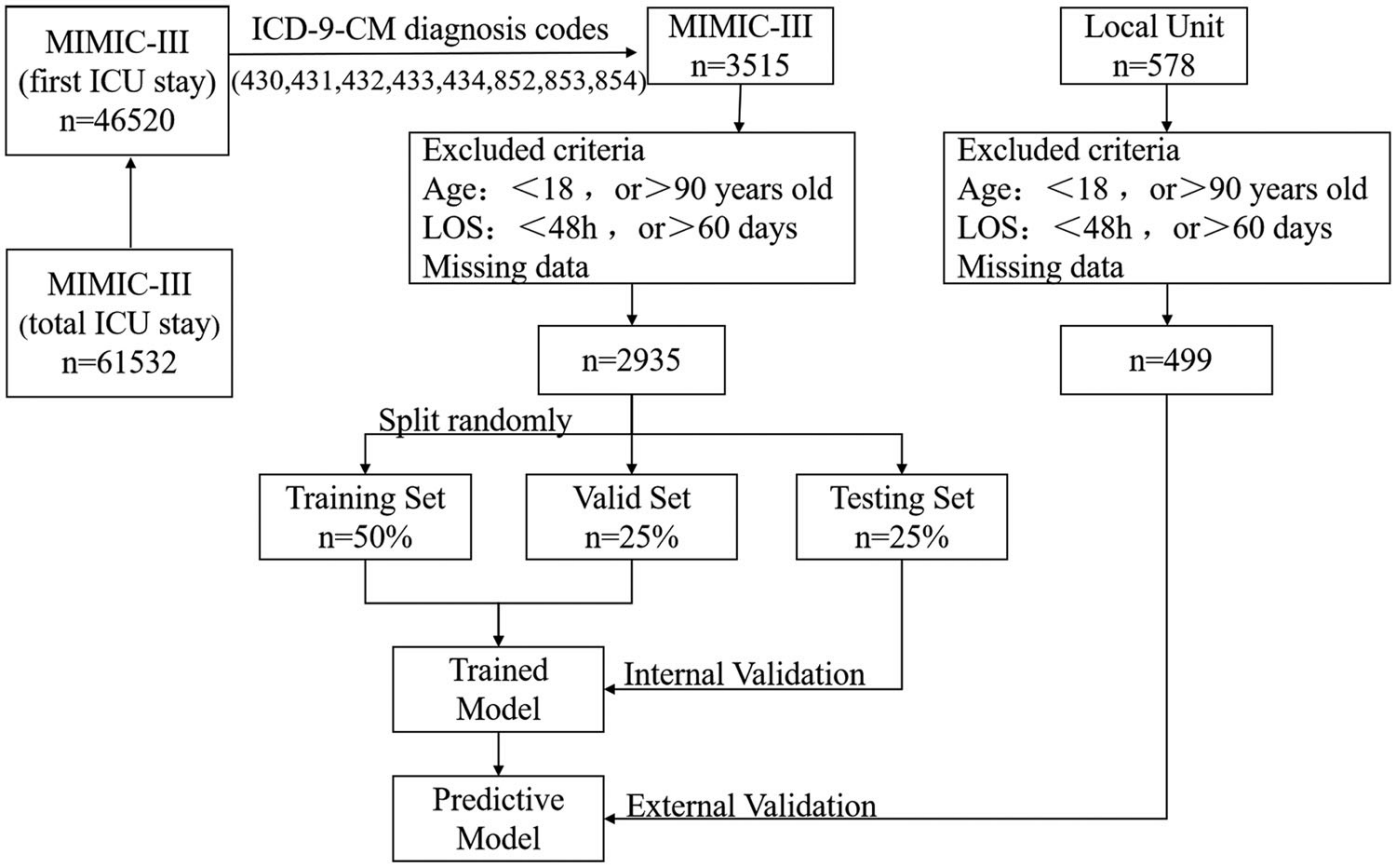
Flowchart depicting the study protocol. MIMIC-III: Medical Information Mart for Intensive Care III; LOS: length of stay; ICD-9-CM: International Classification of Diseases, ninth revision, Clinical Modification.

This was a retrospective study based on two different cohorts: the MIMIC-III database (internal validation) and the database held by the First Affiliated Hospital of Fujian Medical University (external validation). The MIMIC-III database features critical care data for over 40,000 patients admitted to intensive care units at the Beth Israel Deaconess Medical Center (BIDMC) between 2001 and 2012 [18]. The data held by the MIMIC-III database have been de-identified and all patient identifiers have been sealed in accordance with the Health Insurance Portability and Accountability Act (HIPAA) Safe Harbor provision. Researchers can apply to use this database without charge. In the present study, we used MIMIC-III v1.4. To gain access to the database, we completed relevant training courses at the National Institutes of Health and got the certificate (reference: 41733653). We also used another database held by our own hospital (a tertiary teaching hospital); this database featured 499 patients diagnosed with acute cerebrovascular disease and admitted to the ICU between January 2016 and February 2021. This study was approved by the ethics committee of the First Affiliated Hospital of Fujian Medical University (Reference: [2020]280). Figure 1 shows a flowchart depicting the study protocol.

We used International Classification of Diseases, ninth revision, Clinical Modification (ICD-9-CM) diagnosis codes (430,431,432,433,434,852,853,854) to identify eligible patients (acute cerebrovascular disease) for analysis. We excluded patients younger than 18 years of age or more than 90 years of age, those who stayed in the ICU for <48 h or more than 60 days, and those with an incomplete set of laboratory or medical data.

### Definition and staging of AKI

The definition and staging of AKI was based on serum creatinine (SCr) criteria according to the 2012 Kidney Disease: Improving Global Outcomes (KDIGO) guidelines [19]. A diagnosis of AKI could be made if the SCr had increased beyond 0.3 mg/dL (≥26.5 μmol/l) within 48 h, or if the increase of SCr was not less than 1.5-folder different from baseline, and these situations were known or presumed to have occurred within the past seven days.

The staging of AKI was described as follows. AKI stage 1: SCr lay in a range that was 1.5–1.9-fold that of the baseline, or ≥0.3 mg/dL (≥26.5 μmol/L), and these situations were known or presumed to have occurred within the past seven days. AKI stage 2: SCr lay in a range that was 2.0–2.9-fold higher than baseline. AKI stage 3: SCr was not less than 3.0-fold higher than baseline, or the increase of SCr was not less than 4.0 mg/dL (≥353.6 μmol/L), or renal replacement therapy had been initiated.

Normally, the baseline was set as the patient’s initial SCr test prior to ICU admission. However, the data provided by the MIMIC-III database only contained patient data from the period after ICU admission. Therefore, the baseline was set as the minimum value after ICU admission. A patient would be diagnosed with AKI if the SCr level increased and was not less than 1.5-fold higher than the baseline within 48 h. Urine volume was not included in our analysis due to incomplete datasets.

### Data collection

For each of the included patients, we collated data relating to 23 features as follows: (1) demographics (sex, age, length of ICU stay); (2) medications (in accordance with the literature, we selected several factors that are known to exert significant influence on AKI, including diuretics, non-steroidal anti-inflammatory drugs (NSAIDs), vasopressors, aminoglycosides, mannitol, colloid bolus, continuous renal replacement therapy (CRRT) and mechanical ventilation); (3) comorbidities (hypertension, diabetes, and infection); (4) laboratory test results, including white blood count (WBC), hemoglobin (HgB), platelets (PLT), creatinine (CR), blood urea nitrogen (BUN), albumin (Alb), sodium (Na^+^), potassium (K^+^), and bicarbonate (HCO3^−^), and (5) prognosis (whether patients died after discharge). For all parameters, we used the first values obtained during the first 24 h in the ICU.

### Data pre-processing

**Figure 2.**
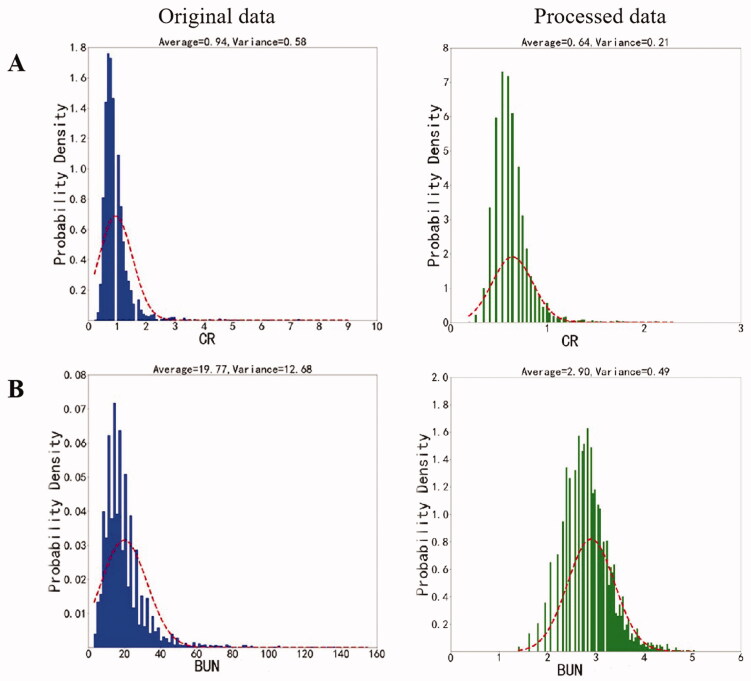
An example of data pre-processing involves the normalization of data. Original data VS Processed data (the data after logarithmic transformations); CR, serum creatinine; BUN, blood urea nitrogen.

We identified and removed duplicate data and used ‘0′ in the analysis from the beginning for any parameters with missing data. We found that the distribution of some parameters was skewed or concentrated within a very small range. To normalize such data, we used logarithmic transformations so that the data were distributed in a manner that was more similar to the Gaussian distribution. Figure 2 shows a typical example of such transformation.

**Figure 3.**
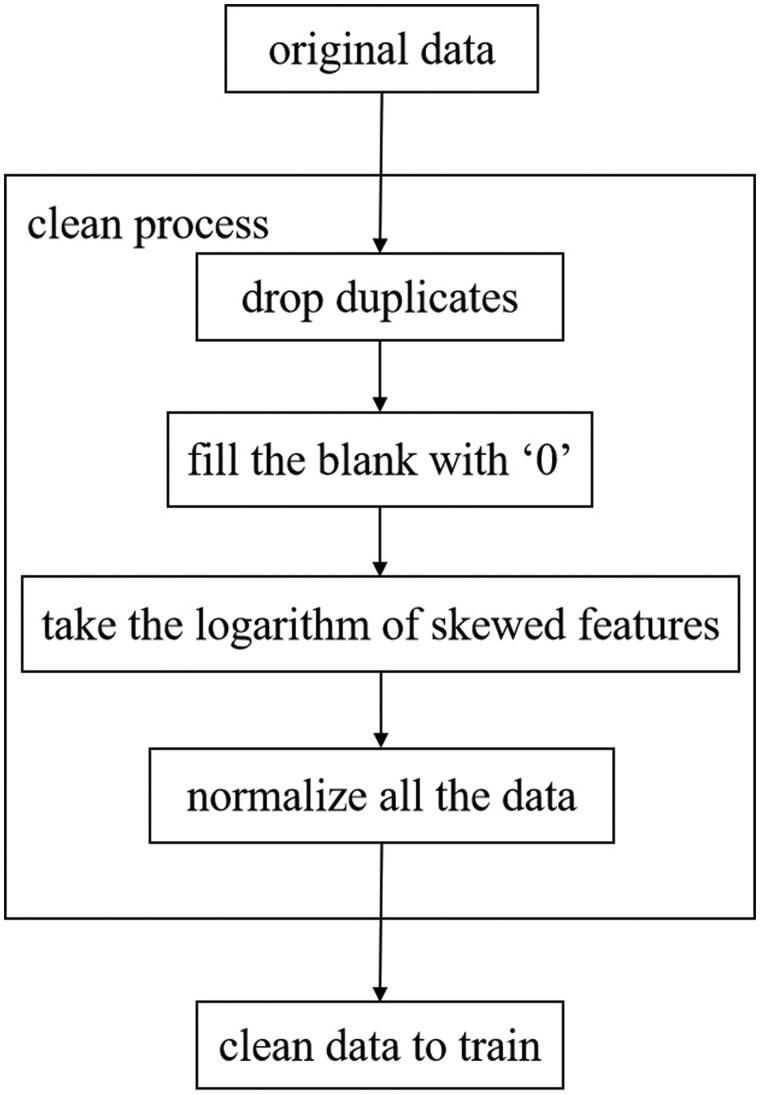
The data cleaning process.

To account for different data ranges for different features, we also normalized data that featured scores and not classifications. We used min-max scaling for data normalization. The min-max scaling transforms the original data into the range of [0,1], and the normalized formula is Xnorm=(X-Xmin)/(Xmax-Xmin). Xnorm is the normalized data, X is the original data, Xmax and Xmin are the maximum and minimum values of the original data respectively. The method can realize an equal scale of the original data. This allowed us to analyze data in a standardized manner. Figure 3 depicts a typical example of data cleaning.

### Predictive model and data split

Simple and unadjusted independent models, such as the Adaboost Decision Tree, were selected in advance, and the relationship between the number of samples and the stability of the model was studied in general. When the number of training samples was lower than 800–1000, we found that the AUROCs (the areas under the receiver operating characteristic curves) and F2 scores improved significantly as the number of samples increased, thus indicating that the number of samples was insufficient originally. When the number of samples exceeded 1200–1400, the degree of improvement was only minor, thus indicating that the sample size was sufficient. Based on this information, the training set featured 1400 samples (and represented 50% of the total sample set).

Following data cleaning, the MIMIC-III database was randomly partitioned into three groups: 50% of samples were used to train the model, 25% of samples were used to validate, and 25% were used as internal tests to complete the basic assessment. We also used our local database to perform external tests, such that we could assess the model in a more comprehensive and objective manner.

In this study, we used five methods to develop risk prediction models for AKI: an extreme gradient boosting (XGBoost), adaptive boosting (AdaBoost), random forest (RF), logistic regression (LR) and multi-layer perception (MLP). A tree-like model represents a flowchart-like structure with each internal node representing a ‘test’ of an attribute. In machine learning, multiple tree-like weak learners would normally be integrated into a model to improve quality. Weak learners are used in a serial manner in the boosting model (XGBoost, AdaBoost) and in a parallel manner in the random forest model; thus, boosting models are updated by each of the weak learners according to the result of the previous one. The random forest model refers to every weak learner by different weightings at the same time. The tree-based supervised machine learning methodology is usually robust when faced with abnormal conditions. Logistic regression is a traditional statistical model while multi-layer perception uses backpropagation techniques for training and can distinguish data that is not linearly separable.

Boosting trees help to reduce bias while bagging trees helps to reduce variance. For each predictive model, we used 5-fold cross-validation to evaluate the performance of these algorithms and employed six popular metrics (AUROC, F2-Score, accuracy, sensitivity, specificity and precision) to evaluate the functionality of these models. We used a loop function to select hyperparameters by grid searching and trained the five models individually using hundreds of combinations for ranking. According to the training log-loss and testing log-loss, we finally selected the best combination for the predictive model.

### Data analysis

Python (3.7.0) was used for all data analyses in this study. For continuous variables, the Shapiro-Wilk test was used to assess whether the data were normally distributed. Data that conformed to the normal distribution were expressed as means ± standard deviation. Comparisons between groups were carried out by the student’s *t* test. Data that were not normally distributed were expressed as medians and interquartile ranges. Comparisons between groups were carried out using the Kruskal-Wallis test. Comparisons between groups were carried out with the Chi-squared test for categorical variables. XGBoost was used to calculate the scores for important features and perform feature selection. The performance of each feature was analyzed and we used a range of factors to evaluate the performance of the model, including the AUROC, F2-Score (F2), accuracy, specificity, sensitivity and precision. By placing more emphasis on false negatives, the F2 score is associated with a higher recall rate than precision. A high precision but low recall rate provides us with an extremely accurate outcome but can miss many instances that are difficult to classify. AUROC was applied to assess model discrimination and a calibration curve was applied to assess model calibration. Statistical significance was defined as *p* < 0.05.

## Results

### A comparison of clinical features across different study cohorts

We finally identified 2935 patients with acute cerebrovascular disease from the MIMIC-III database and 499 patients from our local hospital to develop and validate the AKI risk prediction model. The MIMIC-III database was randomly divided into a training set (50% of patients), a validation set (25% of patients) and an internal test set (25% of patients). There were no statistically significant differences between these three groups in terms of all the features, indicating that the division was random. There was no difference in terms of hypertension, diabetes, SCr, and bicarbonate, although there was a notable difference in terms of other clinical features between the MIMIC-III database and our local database, as shown in Table 1, illustrated that there was a significant difference in these two cohorts. Even with the big difference in these two cohorts, however, our machine learning models can still achieve good performance for the AKI risk prediction, it means that the model could be generalized to other cohorts.

**Table 1.** Characteristics of the study population.

Factors	Training set (*N* = 1467)	Validation set (*N* = 734)	Testing set (*N* = 734)	Total (*N* = 2935)	Local (*N* = 499)	*P* value
Sex (male), *N* (%)	753 (51.3)	373 (50.8)	374 (51.0)	1500 (51.1)	341 (68.3)	<0.001
Age (years)	66.8 (54.1, 78.4)	66.7 (53.0, 78.5)	67.5 (53.8, 78.1)	66.8 (53.6, 78.3)	61.0 (50.0, 71.0)	<0.001
Length of stay in ICU (days)	8.7 (4.7, 15.2)	8.5 (4.7, 15.3)	8.2 (4.8, 14.8)	8.6 (4.7, 15.1)	10.0 (5.0, 18.0)	<0.001
AKI, *N* (%)	268 (18.3)	134 (18.3)	134 (18.3)	536 (18.3)	308 (61.7)	<0.001
Die when discharge, *N* (%)	290 (19.8)	154 (21.0)	150 (20.4)	594 (20.2)	212 (42.5)	<0.001
Hypertension, *N* (%)	862 (58.8)	445 (60.6)	445 (60.6)	1752 (59.7)	304 (60.9)	0.605
Diabetes, *N* (%)	301 (20.5)	154 (21.0)	151 (20.6)	606 (20.6)	95 (19.0)	0.410
CRRT, *N* (%)	12 (0.8)	7 (1.0)	6 (0.8)	25 (0.9)	19 (3.8)	<0.001
Mechanical ventilation, *N* (%)	771 (52.6)	390 (53.1)	393 (53.5)	1554 (52.9)	390 (78.2)	<0.001
Mannitol, *N* (%)	43 (2.9)	22 (3.0)	34 (4.6)	99 (3.4)	368 (73.7)	<0.001
Colloid bolus, *N* (%)	83 (5.7)	39 (5.3)	44 (6.0)	166 (5.7)	373 (74.7)	<0.001
NSAIDS, *N* (%)	405 (27.6)	193 (26.3)	207 (28.2)	805 (27.4)	283 (56.7)	<0.001
Diuretics, *N* (%)	468 (31.9)	224 (30.5)	249 (33.9)	941 (32.1)	412 (82.6)	0.047
Vasoactive drugs, *N* (%)	274 (18.7)	116 (15.8)	123 (16.8)	513 (17.5)	231 (46.3)	<0.001
Aminoglycosides, *N* (%)	235 (16.0)	118 (16.1)	126 (17.2)	479 (16.3)	42 (8.4)	<0.001
Infection, *N* (%)	522 (35.6)	246 (33.5)	248 (33.8)	1016 (34.6)	412 (82.6)	<0.001
White blood cell count (10^9/L)	10.6 (8.2, 13.9)	10.9 (8.3, 13.8)	10.8 (8.0, 13.7)	10.7 (8.2, 13.9)	11.9 (9.5, 16.0)	<0.001
Hemoglobin (g/L)	11.8 (10.4, 13.1)	12.0 (10.6, 13.3)	12.1 (10.6, 13.3)	11.9 (10.5, 13.2)	11.4 (9.6, 13.2)	<0.001
Platelet count (10^9/L)	218.0 (170.0, 276.0)	221.0 (174.0, 282.0)	218.0 (167.0, 274.3)	218.0 (170.0, 277.0)	177.0 (135.0, 234.0)	<0.001
Creatinine (mg/dl)	0.8 (0.7, 1.1)	0.8 (0.7, 1.0)	0.8 (0.7, 1.0)	0.8 (0.7, 1.0)	0.8 (0.6, 1.0)	0.659
Blood urea nitrogen (mmol/L)	17.0 (12.0, 23.0)	17.0 (13.0, 23.0)	17.0 (12.0, 23.0)	17.0 (12.0, 23.0)	8.5 (5.8, 14.7)	<0.001
Potassium (mmol/L)	3.9 (3.6, 4.2)	3.9 (3.6, 4.2)	3.9 (3.6, 4.2)	3.9 (3.6, 4.2)	3.9 (3.5, 4.3)	0.047
Sodium (mmol/L)	140.0 (137.0, 142.0)	139.0(137.0, 142.0)	139.0(137.0, 142.0)	139.0 (137.0, 142.0)	146.4 (140.4, 154.5)	<0.001
Bicarbonate (mmol/L)	25.0 (23.0, 27.0)	25.0 (23.0, 27.0)	25.0 (23.0, 27.0)	25.0 (23.0, 27.0)	24.8 (21.4, 28.4)	0.297
Albumin (g/L)	3.3 (3.0, 3.7)	3.3 (3.1, 3.7)	3.3 (3.0, 3.6)	3.3 (3.0, 3.7)	3.7 (3.3, 4.2)	<0.001

Abbreviations: AKI, acute kidney injury; CRRT, continuous renal replacement therapy; NSAIDS, Non-Steroidal Anti-inflammatory Drugs.

### Scores for important features

**Figure 4.**
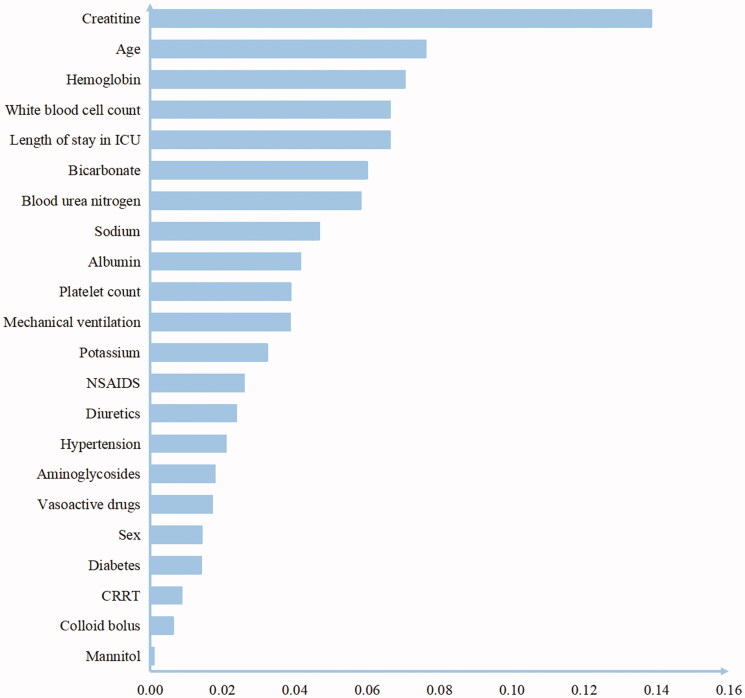
Feature importance scores. Features selected by machine learning methods, ranked by importance scores. Abbreviations and annotations: NSAIDS, Non-Steroidal Anti-inflammatory Drugs; CRRT, continuous renal replacement therapy.

Twenty-three important features were extracted for the risk prediction of AKI. We then investigated the relative importance of each factor for AKI prediction. We used XGBoost, AdaBoost, and random forest classification models to acquire the relative importance score for each feature individually and then took the average from all three models (Figure 4). Laboratory tests CR, HGB, WBC, HCO3, BUN, Na, Alb, and PLT, combined with age and the length of ICU stay, were the top ten most important factors. This result suggested that these factors were strongly associated with AKI, guiding the physicians to pay more attention to these factors.

### Comparison of AUROC values across different models

**Figure 5.**
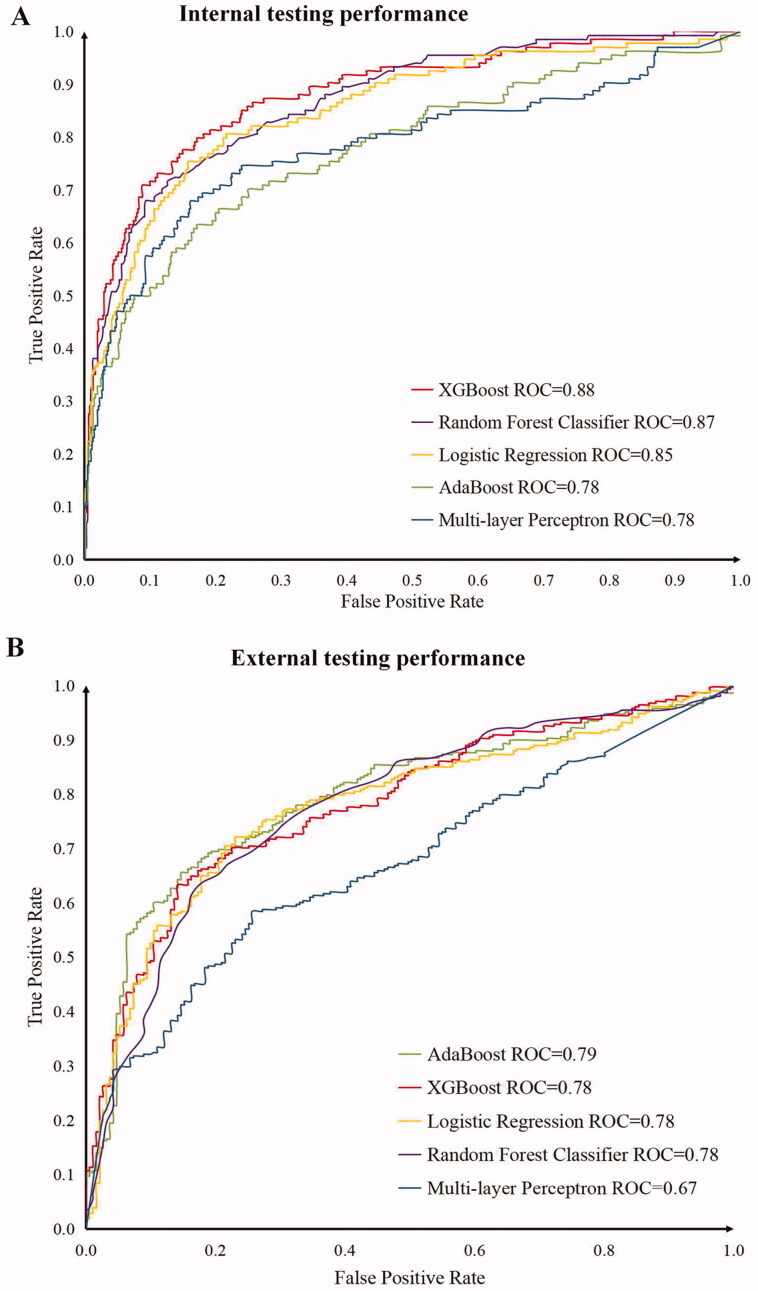
Performances of the prediction models for internal and external validation. (A) Internal testing performance: Receiver operating characteristic curve for the MIMIC-III test cohort. (B) External testing performance: Receiver operating characteristic curve for the local test cohort.

Model discrimination was assessed using AUROC values. Figures 5A–B showed that the performance of different models varied under these two different cohorts. The AUROC values of the XGBoost model were 0.880 (95%*CI*: 0.831–0.929) and 0.780 (95%*CI*: 0.731–0.829) respectively in the internal validation set and external validation set, while the MLP model performed the weakest (0.780, 95%*CI*: 0.725–0.835 and 0.670, 95%*CI*: 0.615–0.724). As we can see, the XGBoost model had higher AUROC values, indicating that the XGBoost model was better at predicting AKI risk in patients with acute cerebrovascular disease than other models. The scores for some models (such as the XGB, RF, LR, and MLP) decreased by 0.070–0.100 from the internal test to the external test, mainly due to the differences between the MIMIC-III database and our local database. However, our local database still showed a sufficiently strong AUROC to demonstrate the model's ability to generalize to other cohorts.

### Calibration curves for internal validation on the MIMIC-III test database

**Figure 6.**
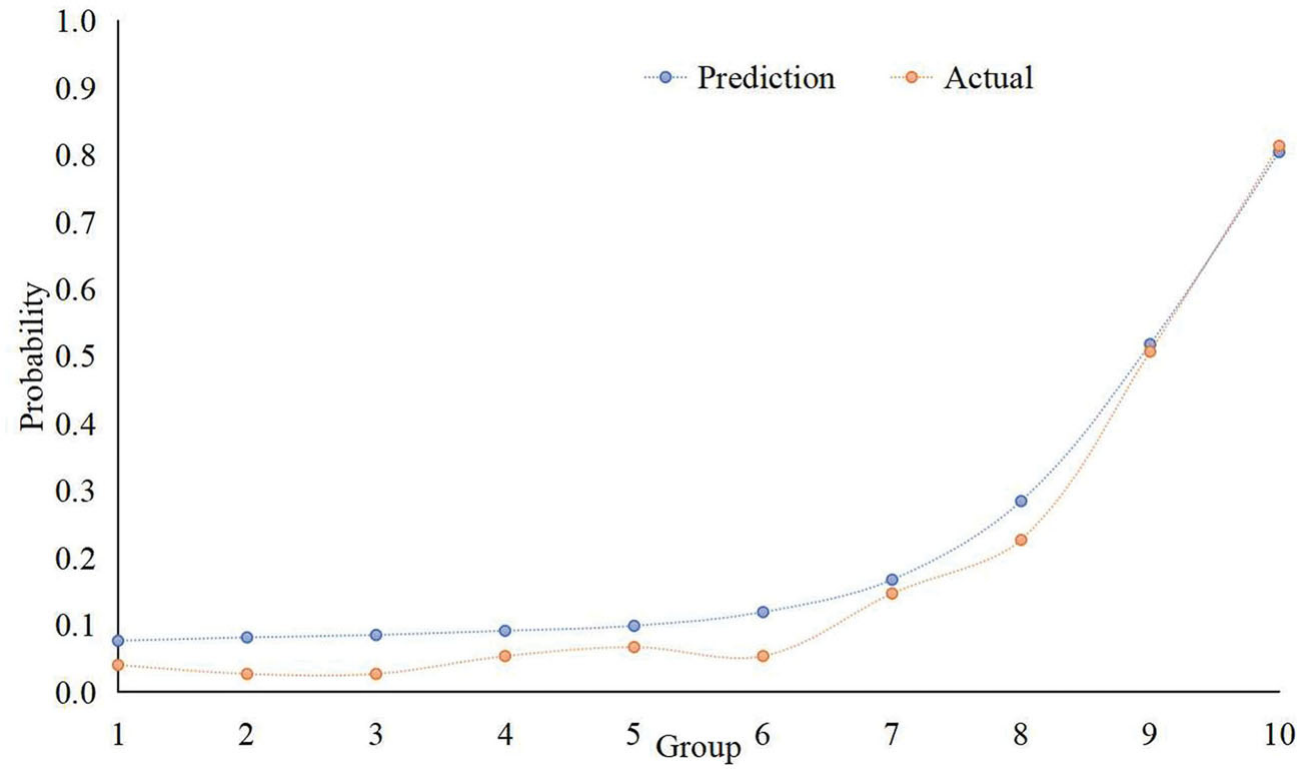
Calibration curves for internal validation on the MIMIC-III test database. The X-axis represented ten subgroups according to the predicted probabilities from 0 to 1. The Y-axis represented the probabilities of the incidence of AKI.

We further performed the calibration curve by the XGBoost model. As shown in Figure 6, the calibration curve indicated the predicted and actual incidence of AKI. The closer the predicted calibration curve was to the actual calibration curve, and the more similar the predicted probabilities were to the actual probabilities. Thus, the result suggested that the calibration in the XGBoost model was better than others.

### A comparison of the performance for different models

Furthermore, six popular metrics (AUROC, F2-Score, accuracy, sensitivity, specificity and precision) were used to evaluate the performance of these models. Comparative analysis of the different models showed that AUROCs and accuracies were both good in general, although F2 Scores were low (Table 2). The main problem was that the recall rate for sensitivity was low. In general, we hoped to achieve an accuracy of >60% when identifying positive patients in the primary screening, and at least 60% of patients were predicted to be positive by the model. On this basis, the higher the AUROC, the better the performance. The XGBoost model had higher AUROC (0.880, 95%*CI*: 0.831–0.929), illustrating that it had better performance at predicting AKI risk in patients with acute cerebrovascular disease than other models.

**Table 2.** The performance of different models based on the MIMIC-III database.

Model	AUROC	F2-Score	Accuracy	Sensitivity	Specificity	Precision
XGB classifier	0.880	0.510	0.879	0.470	0.970	0.778
AdaBoost classifier	0.780	0.420	0.846	0.388	0.948	0.627
Random forest classifier	0.870	0.450	0.872	0.403	0.977	0.794
Logistic regression	0.850	0.680	0.812	0.746	0.827	0.490
Multi-Layer perception	0.780	0.510	0.860	0.485	0.943	0.657

Considering the impact of different data distributions on the model, we did find that the overall score did fall slightly (such as the AUROC and accuracy), although the scores were still acceptable (the AUROC of the XGBoost model was from 0.880 to 0.780 and the accuracy was from 0.879 to 0.713). This indicated that the model did possess a generalization ability that could be applied to different regions and different groups (Table 3).

**Table 3.** The performance of different models was tested by our local database.

Model	AUROC	F2-Score	Accuracy	Sensitivity	Specificity	Precision
XGB classifier	0.780	0.770	0.713	0.769	0.623	0.767
AdaBoost classifier	0.790	0.700	0.729	0.669	0.827	0.862
Random forest classifier	0.780	0.840	0.711	0.870	0.455	0.720
Logistic regression	0.780	0.760	0.739	0.753	0.717	0.811
Multi-Layer perception	0.670	0.560	0.617	0.526	0.764	0.783

## Discussion

AKI is a complex disease associated with poor outcomes. Consequently, it is immensely challenging to develop a simple individual model that could accurately predict the risk of AKI in ICU patients with acute cerebrovascular disease. One previous study reported that the incidence of AKI after all stroke types was 11.6% while subgroup analyses further revealed that the pooled prevalence rate of AKI after acute ischemic stroke was 19.0% and the rate of AKI after intracerebral hemorrhage was 12.9% [20]. In the present study, we showed that the incidence of AKI in critically ill patients with acute cerebrovascular disease in the MIMIC-III database was 18.3%; this concurred with the findings of other studies. Machine learning methods had been successfully used to develop models to predict and to detect AKI for older patients [2]^1^ and to predict mortality in ICU patients with sepsis [22]. Machine learning methods were also successfully established to predict cardiac surgery-associated acute kidney injury (CSA-AKI), which determines risks following cardiac surgery, enabling the optimization of postoperative treatment strategies to minimize the postoperative complications following cardiac surgeries [23]. If we are able to accurately identify patients at high risk of AKI by applying machine learning methods, we may be able to prevent or reduce mortality.

Many factors have been associated with AKI after intracerebral hemorrhage, including age, hypertension, diabetes, hypovolemia, central nervous system injury, nephrotoxic drugs, and infection [24]. The variables we selected to include in our model development were selected carefully from the existing literature. In this study, the new models were trained with the MIMIC-III database and tested for predictive ability by applying data acquired from our local hospital database. Although the two databases were very different, the predictive models still showed good results for both databases.

Using advanced machine learning techniques, we identified the top ten most important factors associated with AKI. We found that laboratory tests (such as CR, HGB, WBC, HCO3^-^, BUN, Na^+^, Alb, PLT) might have strong associations with AKI. This result could be explained by the fact that patients who had anemia, inflammation, metabolic acidosis, hypernatremia, hypoalbuminemia, and a low platelet count, might easily suffer from AKI compared to others. Anemia is associated with an increased risk of AKI; the potential mechanisms responsible for this include reduced renal oxygen delivery, worsening oxidative stress, and impaired hemostasis [25]. AKI is also associated with intrarenal and systemic inflammation. Inflammation is essential for eliminating microbial pathogens and repairing tissue after injury. Leukocytes infiltrate the injured kidneys *via* the circulatory system and induce the generation of inflammatory mediators, such as cytokines and chemokines, thus causing damage to the kidney [26]. These resident and infiltrating leukocytes play an important role in the AKI [27]. Studies have also shown that lower serum bicarbonate levels are an independent risk factor for the development of AKI [28]. It is thought that hypernatremia can result in renal dysfunction *via* intravascular dehydration and vasoconstriction, either directly or through a tubuloglomerular feedback mechanism [29]. Hypoalbuminemia is also a risk factor for AKI; therefore, serum albumin determinations might be useful to identify patients at increased risk for AKI. There are several possible mechanisms underlying these effects, including the expansion of intravascular volume, antioxidant function, the maintenance of renal perfusion and glomerular filtration, thus mitigating the effect of nephrotoxic medications [25,30]. Platelets play a significant role in the coordinated immune response to infection and therefore in the inflammation and coagulation dysfunction that contributes to organ damage during sepsis [31]. In our clinical work, we can easily acquire these parameters and use this information to predict patients at a higher risk of AKI. This is a reminder that we should pay close attention to these indicators in our clinical work.

Our research also showed that some clinical factors (such as age, respiratory failure requiring mechanical ventilation, and the length of ICU stay) were likely to be associated with AKI in ICU patients with acute cerebrovascular disease. Studies have shown that age is a risk factor for acute kidney injury [9,32]. Other studies have shown similar results in that respiratory failure requiring mechanical ventilation was independently associated with AKI [6,21,33]. In other words, critically ill patients, with acute cerebrovascular disease, who require mechanical ventilation, can readily suffer from AKI. Thus, the longer ICU stay, the more severe the disease and the higher the risk of AKI.

It is possible that brain-kidney crosstalk may occur *via* increased sympathetic nervous system activity, distant hormonal communication, and an inflammatory state [34]. Intracerebral hemorrhage may directly affect the function of the hypothalamus and brainstem, thus affecting the regulation of sympathetic nerves, causing the constriction of renal vessels, thus resulting in ischemic necrosis in the renal tubules; ultimately, this will lead to AKI [24]. It is important to prevent and treat AKI in patients with intracerebral hemorrhage to protect renal function as early as possible and avoid risk factors that can cause renal injury, such as mannitol [35]. The reason why CRRT and mannitol received lower importance scores in our analysis may have been due to the small number of patients in our cohorts. Some studies have shown that there is a close correlation between the occurrence of cerebrovascular events and renal injury; these are two conditions that can aggravate each other [36]. A recent study also found predictive nomogram incorporating cystatin C is beneficial for physicians to evaluate possibilities of AKI in patients with traumatic brain injury [37]. These models will help doctors to pay more attention to high-risk patients and anticipate potential complications.

In this study, we used five different machine learning methods (XGBoost, AdaBoost, RF, LR and MLP) to build predictive models. Six popular metrics (AUROC, F2-Score, accuracy, sensitivity, specificity and precision) were used to evaluate the performance of these algorithms. As we know, high specificity values lead to fewer false-positive results, and high sensitivity values lead to fewer false negatives [38]. High specificity cut points (enriching for patients very likely to develop AKI) may be appropriate for interventions that carry some risk (such as empirical volume resuscitation), while high-sensitivity cut points (ensuring that few patients with impending AKI are missed) might be appropriate for more benign interventions (such as serum creatinine monitoring) [39]. In our model, specificity is good, it means that the model can reduce the misdiagnosis rate, but sensitivity is poor, the omission diagnostic rate might increase to some extent. AUROC is an overall measure of a model considering sensitivity and specificity. The advantage of our model is that it is applicable to patients in our local hospital as external validation, while sensitivity, specificity and AUROC values also had good results and it is easy for broad applicability. For these five machine learning methods, the areas under the receiver operating characteristic curves (AUROCs) for AKI in the MIMIC-III database were 0.880, 0.780, 0.870, 0.850, and 0.780 respectively. For external validation, the AUROCs were 0.780, 0.790, 0.780, 0.780, and 0.670, respectively. The analysis demonstrated that the XGBoost had higher AUROC values in both the internal validation set and external validation set, indicating that the XGBoost model was better at predicting AKI risk in patients with acute cerebrovascular disease than other models. The XGBoost model had also been successfully used in the prediction of volume responsiveness in patients with oliguric acute kidney injury in critical care [15]. Therefore, this new algorithm might provide an efficient risk stratification tool in the clinic because of its high levels of accuracy.

This study has several strengths. Firstly, we used the MIMIC-III database, which contains a large population of ICU patients, to train the model and used a database from our local unit to test the model externally. Secondly, we used five machine learning methods and designed an ensemble model to generate the final results in order to achieve better performance. Finally, the factors we selected can be acquired easily in clinical work. Consequently, our final model has strong applications for doctors when making important clinical decisions.

Nevertheless, it should be noted that some limitations exist in our study. Firstly, our local dataset was too small; this means that our results are not generalizable to other populations in other locations. Therefore, further studies will be needed to confirm the validity of our findings. Secondly, the MIMIC-III database does not include Scr levels during the previous three months. Thirdly, we did not consider urinary criteria to diagnose AKI in our study because data relating to hourly urinary output were not readily available; this may have underestimated the incidence of AKI [40]. What is more, we only used the clinical factors in the first 24 h after admission, but AKI is a dynamic disease, that cannot be used to provide a dynamic prediction. Finally, the clinical values were normalized by min-max scaling methods, which could not be observed directly. Therefore, including additional variables and enlarging the sample size for external validation would be beneficial. Our predictive model is capable of providing clinicians an early estimation about the incidence of AKI, but it has a limited ability to give real-time prediction throughout ICU stay duration.

## Conclusions

This study provided a new methodology to identify patients at high risk of AKI in critically ill patients with the acute cerebrovascular disease by applying machine learning methods. Our predictive model might assist physicians to determine which patients have a high AKI risk and who should be given priority for treatment, thus improving patient prognosis. Further epidemiological studies, using advanced machine learning methods, might improve the performance of AKI risk prediction models. Our future studies would prospectively evaluate the effectiveness of our AKI prediction model and systematically check whether it improves the outcome of AKI patients in clinical practice. Our results suggest that machine learning techniques have the potential to improve the prediction of AKI risk models in critical care research.

## Data Availability

The dataset generated and analyzed during the current study is not publicly available but are available from the corresponding author on reasonable request.

## Abbreviations

AKI: Acute kidney injury
AUROC: Area under receiver operating characteristic
KDIGO: Kidney Disease: Improving Global Outcomes
SCr: Serum creatinine
NSAID: Non-steroidal anti-inflammatory drug
ICU: Intensive care unit
LOS: Length of stay
HGB: hemoglobin
WBC: White Blood Cell Count
BUN: blood urea nitrogen
ALB: albumin
PLT: platelet count
